# Spontaneous phenotypic suppression of GacA-defective *Vibrio fischeri* is achieved via mutation of *csrA* and *ihfA*

**DOI:** 10.1186/s12866-015-0509-2

**Published:** 2015-09-16

**Authors:** Randi L. Foxall, Alicia E. Ballok, Ashley Avitabile, Cheryl A. Whistler

**Affiliations:** Department of Molecular, Cellular and Biomedical Sciences, University of New Hampshire, 46 College Road, Durham, NH 03824 USA; Northeast Center for Vibrio Disease and Ecology, University of New Hampshire, Durham, USA; Gradaute Program in Genetics, University of New Hampshire, Durham, USA; Current address: Department of Surgery, Massachusetts General Hospital and Department of Microbiology and Immunobiology, Harvard Medical School, Durham, USA

## Abstract

**Background:**

Symbiosis defective GacA-mutant derivatives of *Vibrio fischeri* are growth impaired thereby creating a selective advantage for growth-enhanced spontaneous suppressors. Suppressors were isolated and characterized for effects of the mutations on *gacA*-mutant defects of growth, siderophore activity and luminescence. The mutations were identified by targeted and whole genome sequencing.

**Results:**

Most mutations that restored multiple phenotypes were non-null mutations that mapped to conserved domains in or altered expression of CsrA, a post-transcriptional regulator that mediates GacA effects in a number of bacterial species. These represent an array of unique mutations compared to those that have been described previously. Different substitutions at the same amino acid residue were identified allowing comparisons of effects such as at the R6 residue, which conferred relative differences in luminescence and siderophore levels. The screen revealed residues not previously identified as critical for function including a single native alanine. Most *csrA* mutations enhanced luminescence more than siderophore activity, which was especially evident for mutations predicted to reduce the amount of CsrA. Although CsrA mutations compensate for many known GacA mutant defects, not all CsrA suppressors restore symbiotic colonization. Phenotypes of a suppressor allele of *ihfA* that encodes one subunit of the integration host factor (IHF) heteroduplex indicated the protein represses siderophore and activates luminescence in a GacA-independent manner.

**Conclusions:**

In addition to its established role in regulation of central metabolism, the CsrA regulator represses luminescence and siderophore as an intermediate of the GacA regulatory hierachy. Siderophore regulation was less sensitive to stoichiometry of CsrA consistent with higher affinity for the targets of this trait. The lack of CsrA null-mutant recovery implied these mutations do not enhance fitness of *gacA* mutants and alluded to this gene being conditionally essential. This study also suggests a role for IHF in the GacA-CsrB-CsrA regulatory cascade by potentially assisting with the binding of repressors of siderohphore and activators of luminescence. As many phosphorelay proteins reduce fitness when mutated, the documented instability used in this screen also highlights a potentially universal and underappreciated problem that, if not identified and strategically avoided, could introduce confounding variability during experimental study of these regulatory pathways.

**Electronic supplementary material:**

The online version of this article (doi:10.1186/s12866-015-0509-2) contains supplementary material, which is available to authorized users.

## Background

During host colonization, bacteria use complex regulatory hierarchies to orchestrate the adaptive changes that are necessary to bypass host defenses and secure nutrients. For many bacterial species, the GacA regulator directs the expression of multiple, disparate traits important for colonization, primarily by activating the transcription of several non-coding CsrB regulatory RNAs that sequester CsrA, a regulator that binds and influences translation or stability of target mRNAs [1–3]. Many defects conferred by mutations in GacA homologs are due to their inability to relieve CsrA repression of key transcripts involved in central metabolism, motility, and virulence [4–9]. For squid light organ symbiotic *Vibrio fischeri*, GacA is required for normal metabolism, motility, siderophore-mediated iron sequestration, and production of the key symbiosis factor luminescence, which provides protective counter-illumination camouflage for its squid host [10, 11]. The metabolic defects of GacS/GacA mutants in culture recently revealed accumulation of citrate as a potential signal for GacS in a cascade whereby GacA activates the expression of *csrB1* and *csrB2* to antagonize CsrA, and linked regulation of metabolisms to luminescence [12]. In the absence of GacA, *V. fischeri* is impaired in the early initiation stages of host colonization, and is unable to achieve robust growth within light organs, thereby only rarely reaching large enough populations to induce low levels of luminescence via quorum sensing [10]. Although the importance of GacA to squid symbiosis has been established and its relation to CsrA appears conserved and has already revealed important insights into the pathway [12], the extent of CsrA interaction with targets in the complex GacA regulatory hierarchy are not yet fully elucidated.

Even though characterization of global regulators like GacA provides an opportunity to simultaneously identify multiple phenotypes involved in colonization and elucidate the signal transduction cascades that direct the complex colonization process, their pleiotropy creates challenges for their study. Even though many defects in *gacA* mutants in culture, including altered luminescence, motility and siderophore production, are not detrimental under most laboratory conditions, *gacA* mutants’ associated growth defects reduce fitness [10, 13] which could provide strong selection for secondary mutations that allow escape from the defect, known as suppression. Depending on whether suppressor mutations dramatically change the appearance or behavior of mutants, they may go unnoticed and undetected without whole genome re-sequencing.

Suppression, when applied in a genetic screen, is a powerful tool for identifying interacting partner proteins, domains of importance in protein activity and specificity, and associated defects that provide biological insight [14]. Here we characterize a high frequency suppression phenomena observed in *gacA* mutants of *V. fischeri* that allowed the characterization of effects of a broader distribution of *csrA* mutations than previously. Genetic analysis of the naturally occurring *gacA* suppressors indicates that the suppressor mutations reduced the amount or function of the CsrA post-transcriptional regulator, consistent with an epistatic relationship of CsrA below GacA in its regulatory hierarchy. The screen also implicates the integration host factor (IHF) heterodimer as a potential mediator of GacA-CsrA regulation. Our results suggest that null mutations in CsrA are not tolerated in *V. fischeri* even in the absence of its regulator, GacA.

## Results and discussion

### Phenotypic suppression of *gacA* mutants occurs through the selective growth advantage conferred by spontaneous *csrA* mutations

**Fig. 1:**
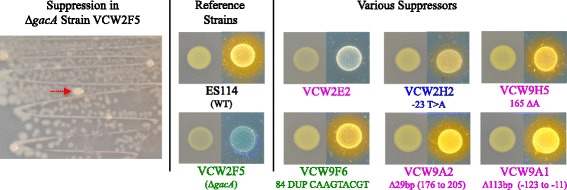
Phenotypic suppression of *gacA* mutants in colony size, opacity and siderophore. Suppression in strain VCW2F5 (*ΔgacA*::Km) is visible on LBS agar as large opaque colonies, a single suppressor is identified by the red arrow, among the small translucent colony-type. Varying degrees of phenotypic suppression in spontaneously arising *gacA* mutant derivatives, compared to reference strains ES114 and VCW2F5, of opacity on LBS agar (*left side*), and siderophore levels observed as an orange halo on CAS agar (*right side*) of each paired picture. The three *gacA* parental strain genotypes are color coded as described in Table 1. *csrA* mutations are indicated below suppressor strains

If the GacA regulatory hierarchy of *V. fischeri* parallels that in other species, pleiotropic defects caused by their inability to antagonize CsrA could be restored by loss of function of *csrA* (VF0538). Unfortunately, our attempts to test this hypothesis by allelic replacement of the native *csrA* gene with several *csrA*-mutant constructs in more than 10,000 recombinants generated in both *gacA*^+^ (wild-type) and *gacA* mutant backgrounds were unsuccessful. This was unexpected given that even difficult-to-achieve mutants with growth defects are typically recovered with screening only a few hundred putative recombinant bacteria [10]. Random transposon mutagenesis also failed to generate a *csrA*::TnErm mutant. Even with the failure of these attempts, several days after growth of Δ*gacA*::Km or *gacA*::TnKm mutants on agar media, suppressors with enhanced growth appeared as larger, yellow and opaque colonies (Fig. 1). These suppressors arose to a high proportion in broth cultures after growth to stationary phase where *gacA* mutants exhibit a severe growth yield defect, but they were rarely detected during exponential growth, consistent with the observation that *gacA* mutants exhibit the same exponential growth rate [10, 15]. Identification of the source mutations conferring spontaneous suppression could reveal intermediates of the GacA regulatory pathway, potentially including CsrA.

Based on the premise that defects in *csrA* could suppress *gacA* mutant phenotypes, especially growth [12], we isolated suppressors that arose in VCW2F5 (Δ*gacA*::Km), VCW2A1 (*gacA*::TnKm) and a suppressor mutant VCW2E2 (*gacA*::TnKm^sup(*ihfAS2*)^) which was identified for its enhanced colony size and opacity on CAS agar (Fig. 1). Strain VCW2E2 was used in this screen to assess whether the unidentified primary suppressor mutation in this derivative would promote the accumulation of a unique distribution of secondary suppressor mutations that would potentially allow us to capture additional informative targets for study. Sixty-four derivatives, including a single derivative (VCW2E3) that was isolated from an unusually luminous *gacA*-colonized squid, were recovered and the *csrA* gene sequenced. All but one derivative with multiple suppressed phenotypes had acquired a mutation in the *csrA* gene indicating most GacA phenotypes are CsrA mediated (Table 1). No *csrA* mutations were identified from 17 derivatives grown under the same conditions that were phenotypically indistinguishable from the parental strain. Sequencing of the *csrB1* and *csrB2* genes [12, 16], encoding the GacA-activiated sRNAs that bind and sequester CsrA [17] in VCW9D1 revealed it harbored a single nucleotide insertion upstream of *csrB2* (Table 1). Consistent with the predicted role of CsrB2 as an antagonist of CsrA, the most probable explanation is that this suppressor mutation enhanced expression of the gene, thereby decreasing CsrA activity. Finally, whole genome re-sequencing of VCW2E2 revealed a single nucleotide substitution within *ihfA* (allele *ihfAS2*) conferring a predicted R60G amino acid substitution that was not harbored by the parental strain (Table 1, See Methods). In agreement with *ihfA* being regulated by CsrA and thus a potential source for suppression, the transcript of this gene co-purified with CsrA in *E.coli* [18]. Once suppressor mutations were identified, probable siblings were excluded from further analysis resulting in a collection of 45 independently derived suppressor mutants, 43 of which mapped to *csrA* (Table 1).

**Table 1:**
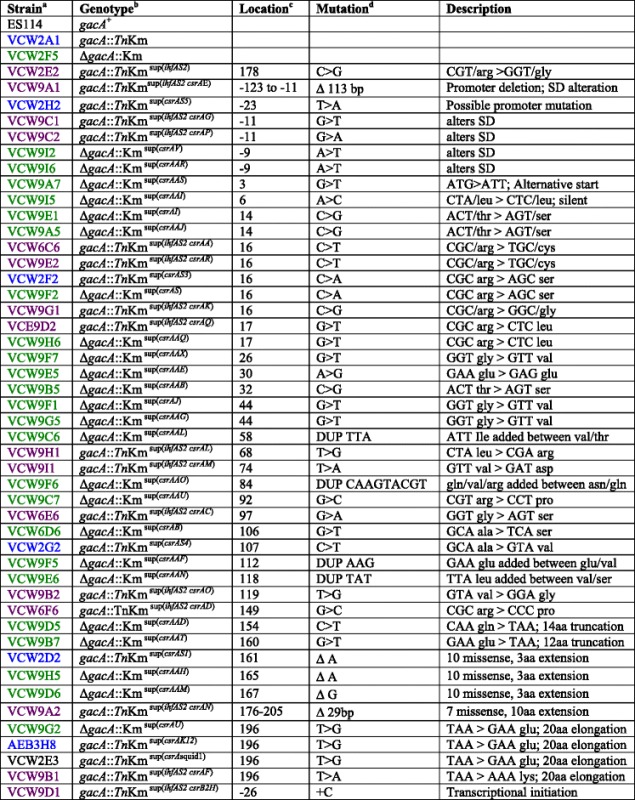
Ancestral and *gacA* mutant suppressors of *Vibrio fischeri* that map to three unique genetic loci

^a^Strain number text is colored for easy identification of derivative *gacA* mutant background, where green denotes ∆*gacA*::Km, blue *gacA*::*Tn*Km, and purple *gacA*::*Tn*Km ^sup(*ihfAS2*)^ strains

^b^Suppressors identified genotypically by superscript “sup” followed in brackets with the gene allele of the identified mutation

^c^Position relative to the first nucleotide of the start codon

^d^DUP; duplication; Δ: deletion; + :insertion of single base

### Distribution of spontaneous *csrA* mutations suggested reduced activity rather than null CsrA mutations enhanced *gacA* mutant fitness

Several informative patterns of acquired mutations in *csrA* became apparent from the distribution and types of spontaneous mutations. Independently-derived identical mutations were recovered at nucleotide position 14, 17 and 44 and all possible substitutions were recovered at position 16, suggesting the screen was near saturation for mutations in *csrA* that provide a fitness benefit (Table 1). The only frame-shift mutations, all generated by single nucleotide deletions, mapped within the sequence encoding the last 10 amino acids which are absent in some naturally occurring CsrA homologs (e.g., RsmA from *Pseudomonas putida* [19]), and each of these were predicted to extend rather than truncate the CsrA protein. No other missense or non-sense mutations that altered or truncated the CsrA protein in these 10 amino acids were recovered, suggesting their loss would not impair CsrA function and thus were not predicted to compensate for the *gacA* mutants’ inability to relieve CsrA repression (Table 1). However, two point mutations that caused truncations of 12 and 14 amino acids were recovered (Table 1). Interestingly, all insertions/duplications maintained the original reading frame. Since frame-shift mutations along with nonsense mutations have a high potential to eliminate protein function (except in the C-terminus, as described) the most plausible explanation for their absence is that null *csrA* mutations did not improve *gacA* mutant fitness. The remaining mutations in the *csrA* ORF were missense point mutations (Table 1).

**Fig. 2:**
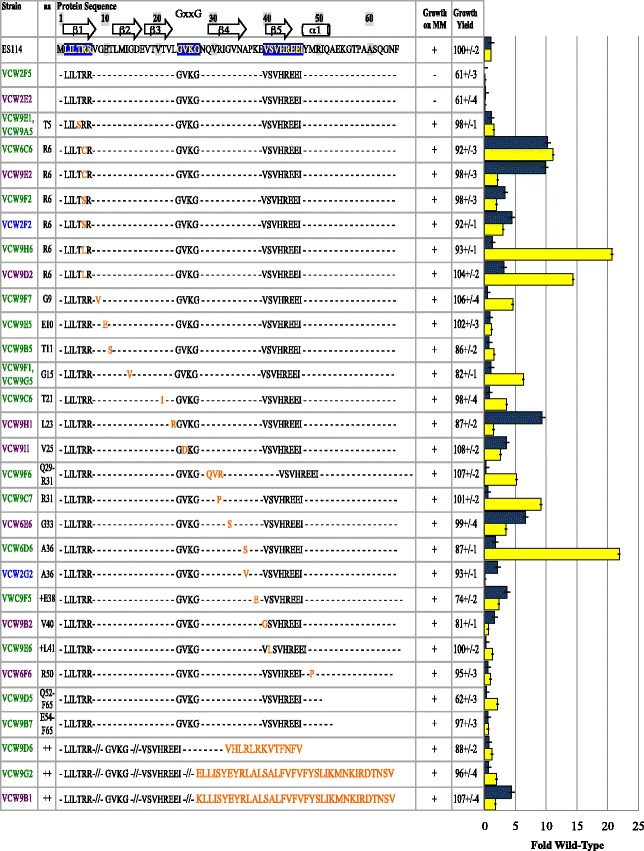
Location of suppressor mutations that mapped to the inferred CsrA protein within conserved regions and the resulting phenotypes relative to wildtype ES114. The native CsrA protein sequence of ES114 was aligned with the secondary structure consensus (β1-5 and α1) and identified conserved sequences [20, 23] that are region 1, the HA RNA binding GxxG motif, and region 2 (*highlighted in blue with yellow text*). For each suppressor the deduced native amino acid (column AA) and predicted amino acid substitution, insertion, deletion or elongation is first identified below the consensus in orange text. For native amino acid position, “+” indicates an insertion of the amino acid described, and a “++” signifies frame shift mutations in every case altering more than 10 aa, and all conferring a protein extension (see Table 1). Growth on minimal agar media which was supplemented with glycerol or N-acetylglucosamine is designated by “+” for growth and “-“no growth. Growth yield (OD_600_) is expressed as a percent of wildtype grown overnight in SWT. Blue bars are percent siderophore levels and yellow bars percent luminescence relative to wildtype, where error bars represent 95 % confidence intervals using 5–8 replicates. The names of the strains are color coded by genetic background as described in Table 1

Many of the mutations mapped to conserved regions consistent with these impairing CsrA function. Several mutations mapped near the conserved HA RNA-binding motif, and one even in the valine in this motif (GVxG), which would agree with previous studies that suggest conservation in this valine is not essential [20, 21]. Twelve mutations mapped to the 2 critical subdomains of conserved amino acids essential to CsrA function (Fig. 2) referred to as region 1 and region 2 [20]. Nine of those mapped to region 1 located in the β1 sheet, and only three to the less conserved region 2 in the β5 sheet (Fig. 2) and these, based on phenotype, only modestly impaired CsrA function as did mutations that caused changes in the C-terminal alpha helix (Fig. 2).

**Fig. 3:**
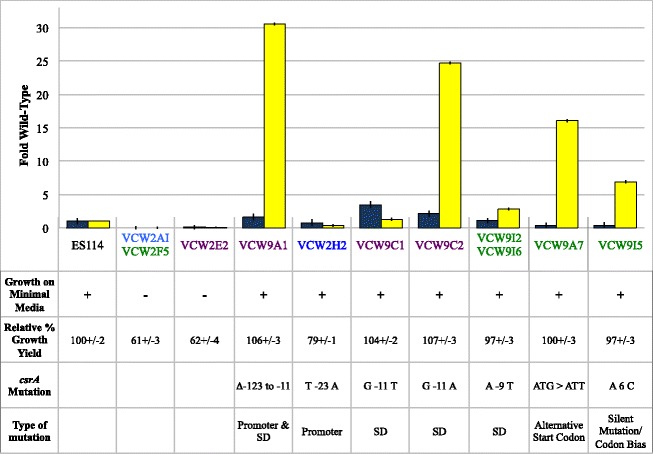
Suppressor mutations predicted to reduce expression of wild-type CsrA. Fold siderophore (*blue bars*) and fold luminescence (*yellow bars*) relative to wild-type ES114 are shown for *gacA* mutants and suppressors, where error bars indicate 95 % confidence intervals of 5–8 replicates. Growth on minimal media agar supplemented with glycerol or N-acetylglucosamine is designated for each strain by “+” for growth and “-“for no growth. Growth yield (OD_600_) is expressed as a percent of wildtype grown overnight in SWT. For each suppressor the *csrA* mutation precedes the row identifying whether a promoter, Shine-Dalgarno (SD), alternative start codon, or silent mutation subject to codon bias [52] is the predicted cause of decreased expression. The names of the strains are color coded by genetic background as described in Table 1

Eight mutations were predicted to change expression, rather than alter function of CsrA (Fig. 3). Among these were a single point mutation that altered the initiation codon from ATG to the documented alternative start codon ATT [22], which could alter the efficiency of translation initiation of a full length *csrA*. Though there is an alternative in-frame start codon downstream of this location, its use would generate a 12 amino acid N-terminal truncated protein and remove previously defined region 1 [20, 23, 24]. One large upstream deletion eliminated the predicted native promoter and altered the Shine-Dalgarno, but semi-quantitative RT-PCR revealed *csrA* transcript was still produced from this mutant (data not shown). Even without its native promoter, the transcript could be expressed from an alternative promoter of the upstream aspartate kinase gene VF_0537. The aggregate data indicated that null mutations in *csrA* were not recovered in this suppressor screen, providing credence that *csrA* mutations are highly deleterious.

### Spontaneous *csrA* mutations typically enhanced luminescence to a greater extent than siderophore

In GacA^−^ derivatives, mutations in *csrA* simultaneously restored growth on the simple sugars, restored growth yield in rich medium, restored siderophore production and often greatly enhanced luminescence (Figs. 2 and 3) [10]. The lack of relative differences for growth yield and utilization of simple sugars contrasts with the highly variable degree of luminescence and siderophore enhancement observed in different suppressors. Furthermore, the relative improvement in luminescence and siderophore activity often did not correlate with each other (Figs. 2 and 3), which has also been reported for different CsrA-regulated traits in *E. coli* [20]. Siderophore activity was on average restored to wild-type levels with a range of 0.2 to 10-fold the wild-type level, and was greater in only 36 % of suppressors. Luminescence much more often surpassed wild-type levels, on average 6-fold, with a minimum of 0.1 and a maximum of 32-fold. Whereas 95 % of the *csrA* mutations fully restored luminescence, only 60 % restored siderophore activity to wild-type levels. The general trend of greater relative increase in luminescence than for siderophore activity with CsrA-impairment may allude to a higher binding affinity of CsrA to regulatory target(s) that govern siderophore: a siderophore defect remained in many suppressors despite mutations that presumably impaired CsrA function. However, other regulatory changes in *gacA* mutants may act synergistically with reduced *csrA* repression in the complex regulation of luminescence. Interestingly, half of the suppressors with 20-fold or more increased luminescence over wild-type harbored mutations that we predict resulted in decreased levels of wild-type CsrA suggesting CsrA binding of mRNA substrate(s) for this trait was especially sensitive to stoichiometry and would agree with a lower affinity (Figs. 2 and 3) although other explanations are possible and this hypothesis may be tested once targets are identified. One mutation that enhanced luminescence more than 24-fold (A36S), did not map to any conserved domain. A valine substitution at this same position that arose in the presence of the *ihfAS2* allele did not enhance luminescence and suggested it only modestly impaired CsrA function, although the *ihfAS2* allele could influence this phenotype separately from CsrA.

### The colony opacity-enhancing *ihfA* mutation increased siderophore and attenuated luminescence of isolates that harbored *csrA* mutant alleles

The primary *ihfAS2* suppressor mutation in strain VCW2E2 had no restorative effect on luminescence, and only slightly increased siderophore production and growth (Fig. 2), but analysis of *gacA csrA* suppressor mutants either harboring or lacking the *ihfAS2* mutation (triple and double mutants respectively) revealed a striking pattern. Most *ihfAS2* mutants surpassed wild type for siderophore activity and all three mutants with a greater increase in siderophore than luminescence levels harbored the *ihfAS2* allele. In support of this conclusion, a suppressor harboring an *ihfAS2* mutation and a *csrA* allele with a predicted R6L substitution (VCW9D2) produced more than twice as much siderophore as a suppressor with the same *csrA* allele without the *ihfAS2* mutation (VCW9H6). Three triple mutants also surpassed wild type for growth yield (Figs. 2 and 3). Luminescence was attenuated in triple mutants compared to double mutants with the same *csrA* alleles (i.e., VCW6C6 and VCW9E2).

As a part of the heterodimeric integration host factor (IHF) protein, IhfA is a global regulator that bends DNA and facilitates recognition and binding of regulatory proteins thereby influences diverse functions [25]. Because the GacA ortholog of *Legionella pneumophila* represses IHF [26], reduced function of IHF does provide a simple explanation for suppression if that hierarchy were conserved in *V. fischeri* and some *gacA* mutant defects were due to overproduction of IHF and enhanced regulatory protein binding. If this hypothesis is true, few defects (e.g., colony translucence) of *gacA* mutants resulted from a lack of *ihfA* repression alone. Alternatively, the spontaneous *ihfAS2* mutation may only modestly alter IHF function, which would be expected for an important global regulator.

The associated phenotypes of the *ihfAS2* allele are consistent with a role for IHF in co-activation of the *lux* luminescence operon, and co-repression of the *iuc* siderophore biosynthetic operon through its ability to enhance the binding of regulatory proteins related to these traits. Because there is an established precedent for IHF mediating activation of *csrB* gene homologs in other species [27, 28], the reduced luminescence of *csrA* mutant strain harboring the *ihfAS2* allele suggests IHF could promote *csrB1* and *csrB2* gene expression and positive regulation of *lux* independently of GacA activation of these genes. However, other mechanisms for IHF activation of *lux* are possible. In *Vibrio vulnificus* IHF works in partnership with the LitR ortholog (SmcR) to activate virulence-associated *vvpE* [26, 29] suggesting IHF activation of luminescence in *V. fischeri* could occur through the promotion of binding of the quorum sensing regulator LitR thereby enhancing expression of LuxR and in turn the *lux* operon. The effect of *ihfAS2* on siderophore activity was consistent with several studies that report transcripts related to iron acquisition are increased by IhfA mutations [26, 30, 31]. The ferric uptake regulator, Fur, and ArcA are the most likely candidates for the co-repression of siderophore with IHF, as other studies report IHF enhancement of aerobic Fur repression, and in the absence of Fur enhancement of anaerobic repression by ArcA [32, 33]. Additional studies to determine whether these or other mechanisms are at play in IHF activation and repression are warranted.

### Newly identified patterns of altered function from comparison of the effects of spontaneous *csrA* mutations with those achieved by alanine scan in CsrA of *Escherichia coli*

A previously applied alanine-scanning mutagenesis that was performed with the *E. coli csrA* gene, used to examine the relative contribution each amino acid residue to CsrA protein function [20], provides an invaluable comparator to our spontaneous suppression screen. Our complementary screen differs in that all potential amino acid substitutions are possible, even the single native alanine residue that was not altered in the alanine scan. In our screen, an A36S substitution caused a 20-fold increase in luminescence and restored siderophore activity indicating the highly conserved A36, positioned into the hydrophobic core of the CsrA homodimer, could be critical to protein folding and 3- dimensional stability [20, 23]. Even the conservative substitution of this alanine with the non-polar and fairly compact valine (VCW2G2) altered CsrA function (Fig. 2). Also, only mutations that improve growth would likely improve fitness. Thus, mutations that map to non-conserved regions that do not contribute to critical protein folding, dimerization, or RNA binding were not recovered, as described previously with the lack of C-terminal truncations. Finally, the screen also dictates that mutations that further impair fitness of the double *gacA csrA* mutant would not be recovered. For example, if the CsrA protein was essential, then none of the spontaneous mutations should eliminate CsrA function. The lack of mutations in highly conserved residues of functional domains, especially for the two guanidine residues of the GxxG HA motif, the absence of frame-shift mutations (except in the last 10 non-essential amino acids), and the detection of a *csrA* transcript in the promoter-deletion mutant with one of most dramatic suppressor phenotypes implies no mutant recovered harbors a null allele and supports our interpretation that CsrA may be essential under the conditions of this screen in *V. fischeri*. Until recently, CsrA was not thought to be essential in most bacterial species [34]; however, among the many published *csrA* mutants, several have insertions near the C-terminus of the protein [35, 36], and such mutations could alter rather than eliminate activity. One report now suggests that CsrA is conditionally essential in *E. coli* [34].

The suppressor screen recovered 8 mutants with mutations that effected the two predicted regions essential to CsrA function, that were confirmed by the alanine scan [20]. Seven were substitutions in amino acids in conserved region 1 that is essential to CsrA function (L2, I3, L4, T5, R6, R7), and a single predicted amino acid insertion in the less conserved region 2 (V40, S41, V42, H43, R44, E45, E46, I47) [20] (Fig. 2). Two possible explanations for the relatively low recovery of suppressor mutations mapping to region 2 are that there are more neutral amino acid substitutions in this more variable region whereas any non-neutral mutations may generate null alleles. Another explanation is that amino acid substitutions in region 2 are well tolerated, and few impair CsrA function enough to confer suppression. Targeted mutagenesis may elucidate whether one of these possibilities is true, although this would be best accomplished through heterologous expression in *E. coli*, given the difficulties in generating a *csrA* null mutation in *V. fischeri* and the impaired growth that multi-copy *csrA* confers of the wild-type ES114 [17].

Suppressors with CsrA mutations in R6 had dramatic phenotypes, implying it is especially critical to CsrA function and is in agreement with the alanine mutagenesis screen in *E.coli* [20]. The CsrA R6A mutation in *E.coli* positively affects expression of all transcripts measured, including highly enhanced *glg*CA and *pga*A expression, and modest increase of *flh*DC expression [20]. To reduce the confounding phenotypes already described, our focus was only on suppressor mutants lacking the *ihfAS2* primary mutation, leaving three different suppressors available for comparison at this site. The R6C substitution enhanced levels of both siderophore and luminescence (~10-fold wild type). Whereas R6L, which is likely a more neutral substitution as both amino acids contain charged side chains, had a similar effect on luminescence, but only restored siderophore activity to wild-type levels; thus, this substitution conferred less impairment on CsrA regulation of siderophore activity. The R6S substitution also resulted in wild-type luminescence implying repression of luminescence was not substantially impaired by this mutation, but siderophore activity increased to 3-fold over wild-type, indicating a greater dysfunction of siderophore repression. That different amino acid substitutions at the same position could influence relative regulation of two different traits, likely through different target transcripts, would not have been revealed by an alanine scan and the basis of these differences warrants further investigation.

In *E.coli,* alanine substitutions in some regions of CsrA had differential influence on certain transcripts, rather than generally affecting function [20]. Substitutions in residues 18–23, and 31–38 differentially affected biofilm formation, and 11–14 and 22 differentially affected glycogen accumulation and motility [18]. Focusing on only mutants lacking the *ihfAS2* mutation, there were five suppressors with CsrA mutations in the biofilm-specific residues and one with a mutation in the glycogen/motility specific residues identified in the *E. coli* CsrA. All six of these mutations resulted in moderate to severe dysfunction of luminescence regulation (wild type levels to 10-fold higher than wild type). Among the phenotype-dependent residues identified, two mutations falling in the biofilm-specific residues, 31–38, were among the nine suppressors with fully restored or increased siderophore activity. Thus, the previously identified biofilm-specific residues appear to contribute substantially to siderophore regulation in *V.fischeri*. Mutations that we propose decreased levels of wild-type CsrA (Fig. 3) and all C-terminal alterations (Fig. 2), had only moderate effects on siderophore activity suggesting these did not interfere as much with siderophore regulation as they did with luminescence regulation.

No suppressors were recovered with CsrA mutations in any of the positions where alanine substitution in *E.coli* affected CsrA levels as would happen if they decreased the stability of the transcript or protein triggering more rapid turnover [20]. Alanine substitutions at positions 18, 20, 34, 52, and 55 lowers CsrA levels, however the mutants have wild-type levels of activity [20]. Our results implied that if substitutions of residues other than alanine at these positions also reduce production, they do not impair function enough to confer suppression. Approximately 16 positions in CsrA cause a decrease in its production when substituted with alanine and also decreased activity [20]. Three suppressors in position 5, 11 and 15 were recovered indicating non-alanine substitutions at these same sites similarly impaired activity.

### A *csrA* suppressor mutation restored squid colonization ability, but only modestly enhanced luminescence *in situ* compared to *in vitro*

To evaluate the ability of the suppressor mutations to restore colonization of and luminescence in squid light organs, two *csrA* suppressors, one that modestly reduced CsrA activity based on its phenotypes (VCW9F6) and one that dramatically reduced CsrA production that also harbored the *ihfAS2* allele (VCW9A1), were evaluated for colonization along with wild-type (ES114), *gacA* mutants (VCW2A1, VCW2F5) and *ihfAS2* suppressor (VCW2E2). The *ΔgacA*, the *gacA*::TnKm^sup(*ihfAS2*)^ and the *gacA*::TnKm^sup(*ihfAS2 csrAE*)^ mutants were unable to colonize squid in two replicate experiments (Table 2). Only wild-type and strain VCW9F6 (*gacA*::TnKm^sup(*ihfAS2 csrAAO*)^) with modestly reduced CsrA function and no *ihfAS2* mutation colonized squid and achieved the same colonization rate assessed at 24 h. These data indicate that the *ihfA* mutation alone does not restore phenotypes responsible for squid colonization deficiencies conferred by the *gacA*::TnKm mutation, but may also allude to IhfA function in colonization. Certainly, the observed luminescence attenuation could impact sustained colonization, although this trait is not necessary for initiation measured here [37]. But, an IHF binding site is predicted in the *syp* locus, which is responsible for the symbiotic polysaccharide, an essential component of a successful initiation, suggesting a possible link of this regulator to squid colonization [38]. Elucidation of the role of IHF in colonization is certainly warranted by this study. The inability of the suppressor that dramatically reduced CsrA function to colonize could imply that even though GacA likely antagonizes CsrA, its activity is important during some of the colonization process. That only the modestly suppressed *gacA* mutant (VCW9F6) was able to colonize squid and only a weak suppressor allele has been recovered from squid light organs (VCW2E3) provides some support for this interpretation in that these mutants retain relatively more native CsrA function based on their *in vitro* phenotypes (Fig. 2). Other unique suppressors from light organ populations were not available for comparison, as they were not recovered over the ten year period of this study. This was likely in part due to care with preparing inoculum once the suppression phenomenon was observed. But given the ease at which suppressors are derived and recovered in culture, their absence from animal studies does agree with the conclusion that CsrA plays a positive role during colonization. Finally, even though strain VCW9F6 has ~4 times greater luminescence than ES114 in culture, it has only modestly, and not significantly, enhanced in luminescence in light organs (Table 2). That loss of CsrA does not enhance luminescence to the same degree in light organs that it does in culture suggested CsrA is repressed in squid compared to culture.

**Table 2:**
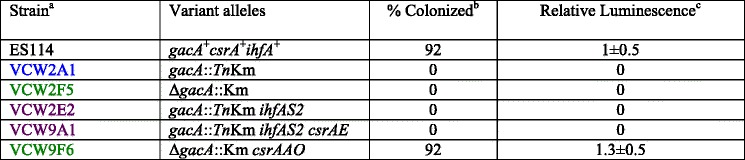
Ability of suppressor mutations to restore colonization and luminescence *in situ*

^a^Strain number text is colored for easy identification of derivative *gacA* mutant background as in Table 1

^b^The combined data from two replicate experiments containing a total of 13 squid for each treatment is reported where colonization was determined by detectable luminescence, and confirmed by destructive plating of light organ homogenates

^c^Relative luminescence (RL) is the luminescence/CFU of each individual squid normalized to the average value of all ES114 colonized squid ± SE

## Conclusions

The CsrA homologs have been described in many bacterial species where they affect behaviors essential for host association, as now inferred in *V. fischeri* as an intermediary of GacA regulation of multiple symbiosis-related traits [9, 12, 17, 39]. Particularly focused *in vitro* analysis of the CsrA protein in a few target species such as *E. coli* has provided insight into structure-function of this post-transcriptional regulator [19]. This study complements those in that the nature of the selective screen allowed the evaluation of effects of a wider array of accessible mutations that alter function and expression. We expect several mutations identified in this screen will provide targets of interest for further analysis in other species where full null alleles can be generated, which has not yet been possible in *V. fischeri*. Although those other systems facilitate such analysis, there are still limitations for extending the *in vitro* analysis in model organisms such as *E. coli* to biologically relevant models for animal infection where these regulatory cascades play a role. For *V. fischeri*, the traits under control of the GacA-CsrA cascade facilitated the colonization process of an accessible host, and revealed subtle differences (e.g., luminescence) in the host compared to *in vitro* analysis. Expanded analysis will likely identify new regulatory partners and mediators of the cascade, such as IHF identified in this screen. Upon identification of the regulatory targets of CsrA that controlled siderophore activity and luminescence, both of which are established as important in this symbiotic model, the potential differences in affinity for targets may be elucidated both *in vitro* and *in vivo*. Thus, this study sets the stage for further analysis of the regulatory hierarchy and regulation in a natural host context that cannot be fully extrapolated from *in vitro* results alone but at the same time, builds upon the wealth of knowledge gained from its study in model laboratory systems.

## Methods

### Bacterial strains and growth conditions

Two previously described *gacA* mutants generated in the squid symbiotic strain ES114, VCW2A1 and VCW2F5 [10], were used for isolation of suppressors (Table 1). *Eschericia coli* strains for cloning and plasmid propagation included DH5α, and Top10 (for pCR2.1TOPO) (Invitrogen). *V. fischeri* was grown in SWT [40], LBS [41], or minimal media derived from CAS solid agar [42] at 28 °C. Media were occasionally supplemented with kanamycin (Km) at 50 μg/ml or chloramphenicol (Ch) at 25 μg/ml for *E. coli* or 2.5 μg/ml for *V. fischeri* to hold selection on the presence of resistance cassettes.

### Directed mutagenesis

Directed mutagenesis of *csrA* (VF0538) was attempted through several standard methods, including those that utilize homologous recombination as described previously [43]. A deletion construct was generated by fusing 1.2 kb of the upstream sequence to 0.3 kb of the downstream sequence of *csrA* by PCR SOE (Table 3) [44]. Although having only 300 bp of DNA flanking one side of the deletion likely reduces efficiency of recombination, other mutants have been successfully generated with less than 200 bp of flanking DNA [10]. This strategy was necessary due to the presence of multiple tRNA genes immediately downstream of *csrA*. The presence of these highly repetitive sequences in an earlier construct facilitated recombination of the plasmid at a number of incorrect sites in the *V. fischeri* genome. Following successful deletion of *csrA* in the amplicon, the PCR product was subcloned into a suicide vector, pEVS79 [43] generating plasmid pAEB1A3. A second construct was made where Km^R^ from pMKm [45] was inserted into a single EcoRI site immediately upstream of *csrA* generating plasmid pAEB3A6 which would aid in selection of mutants having undergone allele replacement. The mutations were introduced into the genomes of wild-type *V. fischeri* and the *gacA* mutant by conjugation and derivatives with single crossovers were isolated by plating on LBS Ch grown for 24 h at room temperature, which enriches for *V. fischeri*. Individual colonies of *V. fischeri* were streak purified onto LBS Ch for plasmid pAEB1A3, or LBS Km for plasmid pAEB3A6 recombinants, and the location of the crossover on either side of the *csrA* gene determined by PCR using primers RsmAF3 and RsmAR4 (Table 3). Derivatives with crossovers within the 300 bp (downstream) flanking DNA were then grown without selection allowing second crossovers to occur. Derivatives of pAEB1A3 plated on LBS, and derivatives of pAEB3A6 plated on LBS Km, were then screened for loss of Ch^R^ by replica plating indicating a second crossover event. Putative mutants (Ch^S^) were then screened by PCR for gene deletion using primers RsmAF3 and RsmAR4 (Table 3). In 5200 mutants evaluated, no derivative harboring the *csrA* deletion was ever identified.

**Table 3:** Oligonucleotide primers used for amplification in PCR

Primer	Sequence (5′ - 3′)	Annealing (°C)	Source
CsrA SOE A	CGCATTCAAGCGGAAACAGCAAAGAATGGCG	50	This Study
CsrA SOE B	CGCCATTCTTTGCTGTTTCCGCTTGAATGCG	50	This Study
CsrA SOE C	AGGTCCCTAGCGATAAGCCGGCTTCGCAAGGTA	50	This Study
CsrA SOE D	TACCTTGCGAAGCCGGCTTATCGCTAGGGACCT	50	This Study
RmsAF3	ATCACTAACCGCTGACCAAG	50	[17]
RmsAR4	AGCCAGGTACTCTATCCAGC	50	[17]
RmsAR7	TCACCGTCTTCAGGAGGC	50	[17]
Kan-2 FP-1	ACCTACAACAAAGCTCTCATCAACC	50	(Epicentre)
RmsAF8	TGTCGGTTATCGTAAATCAAGC	54	[17]
RmsAR9	AACCGTCTATGAAACGACCA	54	[17]
RsmB1F	AGTCAAAAGCGTAGT CTTATTGG	54	[17]
RsmB1R	TCACTGAGGAGAAATGTAACCG	54	[17]
RsmB2F	CTTACAAGCGAGTGAGATTTAGCG	56	[17]
RsmB2R	AGAGGAGGAACTGTATTGTGAGC	56	[17]
GacA4F	TAAATCGGAGTGTCAGTTGTG	51	[17]
GacA5R	AGGAAGGCACTACAGCGTC	51	[17]
QRpoBF1	CAAGAAGTAGATATTGCTGCTCTGT	57	This Study
QRpoBR2	AAGATTGGTGTAGCGATTGGTAA	57	This Study
QCsrAF1	CTGATGATTGGTGACGAAGGA	57	This Study
QCsrAR2	CGCATATAAATCTCTTCACGGTG	57	This Study

After consideration that null mutations harbored by these constructs may be lethal, we attempted to introduce a transposon in the extreme C-terminus of *csrA* at nucleotide 148 as confirmed by sequencing using the EZ::TN™ < Kan-2 > Insertion Kit following the manufacturers protocols (Epicentre) generating plasmid (pJCM24). The transposon was inserted at five amino acids downstream of the mutation generated in *E. coli* [35]. Conjugation and isolation of single crossover derivatives was done, and confirmed by PCR using primers RsmAF3 and RsmAR7 with Kan-2 FP-1 (Table 3). Following second crossover events and loss of the previously integrated mutagenic plasmid, PCR screening with primers RsmAF3 and RsmAR7 with Kan fp-1 (Table 3) of 5000 Km^r^ Ch^s^ derivatives revealed no *csrA*::TnKm mutants were ever generated.

### Isolation and identification of spontaneous suppressors

Spontaneous GacA suppressor mutants harboring unknown mutations were isolated from late log and stationary phase cultures of either the Δ*gacA* mutant or *gacA::*TnKm that were subsequently plated onto LBS or CAS agar and allowed to incubate for approximately 72 h. Large, opaque colonies on LBS or CAS, or siderophore producing colonies with orange halos on CAS agar were isolation streaked onto LBS agar, individual colonies grown in LBS to md-log phase, and immediately stored in 15 % glycerol at −80 °C. For all derivatives restored in one or more phenotype, DNA was extracted [46] and the *csrA* gene was amplified using Expand HiFi polymerase (Roche) using *csrA*-specific forward (RmsAF8) and reverse (RmsAR9) primers (Table 3) and the PCR product directly sequenced by the Hubbard Center for Genome Studies at the University of New Hampshire. The sequenced *csrA* genes from these *gacA*^sup^ mutant suppressors were compared to that of *csrA* from the published genome of *V. fischeri* [47] in order to identify the location of the mutation. Probable siblings were identified by determining if two mutants that harbored an identical mutation were isolated from the same overnight seed culture used to select for suppression; only one such mutant was identified, and removed from further characterization. For the two suppressors that did not harbor mutations in the *csrA* gene, the *csrB1* and *csrB2* genes were amplified with gene specific primers: RsmB1F and RsmB1R for *csrB1*, and RsmB2F and RsmB2R for *csrB2* (Table 3).

### Identification of the *ihfAS2* mutation

Genomic DNA was extracted from ES114, and VCW2E2 late log cultures grown in LBS using the Wizard Genomic DNA Purification Kit and supplied manufacturer protocol (Promega, WI, USA). The DNA quality was assessed visually by electrophoresis. Sequencing libraries were generated from 1 μg of genomic DNA as determined using the Qubit 2.0 fluorimeter (LifeTech, CA, US). DNA was sheared on the Covaris M220 Ultasonicator to a mean size of 500 bp. Libraries were generated using the TruSeq Kit and targeted size selection of 500 bp was completed using the optional gel-extraction method in the TruSeq protocol (Illumina). Genomes were sequenced by a high output mode run 101 bp paired-end reads using an Illumina HiSeq 2500 at the Hubbard Center for Genome Studies at the University of New Hampshire. The Illumina reads were aligned to the published reference ES114 strain (PRJNA58163, assembly GCA_000011805.1) using Breseq pipeline [48]. The predicted mutations (Additional file 1) were cross-referenced with ~60 ES114 (VCW1C6) derivatives also re-sequenced using Illumina HiSeq 2500 101 bp paired-end reads, which revealed differences from the published ES114 reference genome (Additional file 1). Each identified mutant allele was compared to orthologous loci in various *Vibrio* species using BLAST [49] and the nucleotide nr database, which indicated in each instance that the genomic variation in VCW1C6 represented wild-type and likely ancestral, not mutant alleles suggesting they represent errors from the original ES114 genome assembly. For all remaining variant loci identified in VCW2E2 not present in ES114 (VCW1C6) oligonucleotide primers were designed flanking the mutation (Additional file 1) and Phusion High-Fidelity DNA Polymerase (Life Technologies) used to amplify regions by PCR in both VCW2E2 and its parental strain VCW2A1 using an initial denaturation of 1 min at 94 °C, followed by 30 cycles of denaturation of 1 min 94 °C, annealing at described temperature (Table 3) for 1 min, followed by elongation for 1 min at 68 °C. The amplicons were sequenced by the Sanger method [50] using both forward and reverse primers at the Hubbard Center for Genome Studies at the University of New Hampshire. The sequencing reads were aligned with ES114 (BioProject PRJNA58163, assembly GCA_000011805.1) using SeqMan Pro (DNASTAR, Inc. Madison, WI) which allowed confirmation that the single nucleotide polymorphism C > G at base pair position 178 in *ihfA* as the suppressor mutation in VCW2E2 (BioProject PRJNA283549)*.* All other sequences in VCW2E2 were identical to the reference, and were not considered as potential suppressor mutations.

### Semi-quantitative assessment of *csrA* transcripts

For one suppressor whose promoter was presumably deleted, and which exhibited a strong suppressor phenotype, we determined whether the *csrA* transcript was indeed produced by semi-quantitative PCR using the *rpoB* transcript as a housekeeping control gene. Primers amplifying *rpoB* (QRpoBF1 and QRpoBR2) and *csrA* (QCsrAF1, and QCsrAR2) were designed to produce amplicons ~100 bp in length (Table 3). Total RNA was extracted from two biological replicates of late-log cultures of ES114, VCW2A1 (*gacA*::TnKm), and VCW9A1 (*gacA*::TnKm^sup(*ihfAS2 csrA*E)^) grown in SWT. Cells were pelleted and suspended in half the volume of RNA protect (Ambion) and held overnight at 4 °C. RNA was extracted using the UltraClean Microbial RNA Isolation Kit (MoBio Laboratories) followed by DNase treatment with the RTS-DNase kit (MoBio Laboratories). Absence of contaminating DNA was confirmed by end-point PCR using 1 μl of template. The RNA quantity and purity was determined spectrophotometrically using a NanoDrop 2000, and the quality and integrity of RNA assessed by agarose gel electrophoresis. cDNA was synthesized from 500 ng of total RNA using the qScript™ Flex cDNA Synthesis Kit (Quanta Biosciences, MD, US) using 0.5 μM each gene-specific reverse primer and 5 % final volume qScript Reverse Transcriptase at 42 °C for 1 h following the manufacturers protocols. The resulting cDNA was diluted 1/5 with nuclease free water, and immediately used for assays. Assays were performed on 1 μl of cDNA template, in triplicate in 10 μl reactions using using AccuStart PCR Supermix (Quanta Biosciences, MD, US) in separate reactions for *csrA* and *rpoB* cycled at the same time for 30 cycles with a 1 min denaturation at of 94 °C, a 1 min annealing at 57 °C, and a 30 s elongation at of 72 °C. Two replicate RT reactions for each biological replicate RNA were performed. The production of amplicons was assessed at 10, 15, 20, 25, and 30 cycles by electrophoresis of 5 μl of PCR reaction and the presence of amplicons of the correct size visualized after separation with 1.2 % agarose gel electrophoresis in 1x TAE and 1x Gel Red (Phenix Research). Amplicons for *rpoB* were produced from all strains by 20–25 cycles, and from *csrA* between 20 and 30 cycles.

### Phenotypic analysis

All phenotypic analyses were conducted as outlined previously [10]. Briefly, luminescence and growth was determined from cultures grown at 28 °C with shaking (200 RPM) in SWT. Luminescence and density (OD_600_) were measured from 1 ml of culture using a luminometer (Turner Designs) and a spectrophotometer (Eppendorf). LPS was observed as difference in colony opacity on LBS plates. Growth on simple sugars was assessed as cell density in minimal medium supplemented with 32.6 mM glycerol or with 32.6 mM glycerol and 0.5 % casamino acids as a control. Growth yield OD_600_ was measured from cultures grown in SWT overnight (18 h) at 28 °C. The experiments contained 3 replicates and were repeated at least 2 times. Data shown is from a representative experiment and error is expresses as SE. Siderophore production was measured using a modified liquid chrome azurol S (CAS) liquid assay [51]. Briefly, Cultures were grown in minimal medium based on that used for CAS agar plates [30], and grown for eight hours with shaking. The density (OD_600_) was measured and cells removed by pelleting. Equal volumes of the culture supernatant and a CAS assay solution were mixed along with 1 % of shuttle solution (0.2 M 5-Sulfosalicylic acid). After 20 min the absorbance at 630 nm of these mixtures was measured using a plate reader (Tecan). Siderophore is reported percent siderophore units (compared to the blank control) per OD_600_.

Squid colonization was performed exactly as outlined previously [10, 15]. Luminescence was determined with a luminometer (Turner Designs) and colonization by homogenization and plating of squid first frozen at −80 °C at 12, 24 and 48 h after hatching [10, 15]. Luminescence of strains in light organs is reported relative to the average lum/CFU of ES114 colonized squid.

## Declarations

### Availability of supporting data

The data sets supporting the results of this article are included within the article and its additional files. The draft genome sequencing project of *V. fischeri* VCW2E2 is has been deposited with NCBI Genomes under the BioProject PRJNA283549.

### Ethics statement

Invertebrate animal studies to assess the mutualistic bacterial colonization of juvenile squid conducted herein are not regulated in the United States therefore the Institutional Animal Care and Use Committee has no authority for review of such protocols. In lieu of these not falling under the jurisdiction of regulation in the United States, the authors hereby state that these studies were conducted with sincere effort towards the ethical use, care, and treatment of these animals, the number of individuals was minimized and these efforts are consistent with the Basal Declaration (http://www.basel-declaration.org/basel-declaration/) and the International Council for Laboratory Animal Science (ICLAS) ethics guidelines.

## Additional file

Additional file 1:
**The predicted mutations in VCW2E2 when Aligned to ES114 reference strain.** Predicted mutation of strain VCW2E2 compared to the reference identified as genomic position by sequence ID and nucleotide position, followed by the nucleotide change, annotation of allele variation and gene and gene description of gene affected. Evidence of ancestral allele, if applicable, is provided by a list of the top Basic Local Alignment Search Tool results [49] to the allele variant. If no evidence exists, a description of whether the allele was confirmed as mutated or not confirmed is provided. Primers along with annealing temperatures and elongation times for Sanger re-sequencing of the alleles are listed. (XLSX 35 kb)
